# Insight on the Role of Leptin: A Bridge from Obesity to Breast Cancer

**DOI:** 10.3390/biom12101394

**Published:** 2022-09-29

**Authors:** Roberto Buonaiuto, Fabiana Napolitano, Sara Parola, Pietro De Placido, Valeria Forestieri, Giovanna Pecoraro, Alberto Servetto, Luigi Formisano, Pietro Formisano, Mario Giuliano, Grazia Arpino, Sabino De Placido, Carmine De Angelis

**Affiliations:** 1 Department of Clinical Medicine and Surgery, University of Naples Federico II, 80131 Naples, Italy; 2 Department of Translational Medicine, University of Naples Federico II, 80131 Naples, Italy

**Keywords:** leptin, LEPR, obesity, breast cancer

## Abstract

Leptin is a peptide hormone, mainly known for its role as a mediator of adipose tissue endocrine functions, such as appetite control and energy homeostasis. In addition, leptin signaling is involved in several physiological processes as modulation of innate and adaptive immune responses and regulation of sex hormone levels. When adipose tissue expands, an imbalance of adipokines secretion may occur and increasing leptin levels contribute to promoting a chronic inflammatory state, which is largely acknowledged as a hallmark of cancer. Indeed, upon binding its receptor (LEPR), leptin activates several oncogenic pathways, such as JAK/STAT, MAPK, and PI3K/AKT, and seems to affect cancer immune response by inducing a proinflammatory immune polarization and eventually enhancing T-cell exhaustion. In particular, obesity-associated hyperleptinemia has been related to breast cancer risk development, although the underlying mechanism is yet to be completely clarified and needs to be deemed in light of multiple variables, such as menopausal state and immune response. The aim of this review is to provide an overview of the potential role of leptin as a bridge between obesity and breast cancer and to establish the physio-pathological basis of the linkage between these major health concerns in order to identify appropriate and novel therapeutic strategies to adopt in daily clinical practice.

## 1. Introduction

Breast cancer (BC) represents the most frequently diagnosed tumor in women worldwide, with an estimated incidence of 2.3 million new cases in 2021 [1]. During the past decades, BC incidence has markedly increased, reflecting both the improved detection strategy and the rising prevalence of modifiable environmental risk factors, especially in developed western countries [2]. Indeed, lifestyle habits such as alcohol intake, physical activity, body fatness, and weight gain have been largely reported to impact cancer risk development [3]. In particular, obesity, nowadays considered a global epidemic, is a well-known predisposition state for several pathologic conditions. According to the World Health Organization (WHO), obesity prevalence has nearly tripled since 1975: 650 million people, aged 18 or over, are affected by obesity, and it is estimated to hit 1 in 5 adults by 2025 [4]. Furthermore, the correlation between cancer development and obesity has been widely accepted, although the underlying physio-pathological mechanisms have not been completely established yet [5].

The adipose tissue represents the body’s largest endocrine organ and maintains homeostasis through the secretion of multiple cytokines, chemokines, and growth factors [6]. Lean adipose tissue activity is normally balanced between proinflammatory and anti-inflammatory mediators. Eventually, this balance may be lost as a result of the adipose tissue expansion associated with obesity [7]. Adipocytes’ hyperplasia and hypertrophy provoke activation of adipose tissue macrophages (ATMs), which leads to recruitment of inflammatory cells, as mast cells, and CD8-positive T lymphocytes, through the secretion of chemokines, such as monocyte chemoattractant protein-1 (MCP-1) [8]. Therefore, the hypertrophic adipocytes are surrounded by ATMs, thus resulting in the formation of typical crown-like structures (CLS) [9,10,11], considered a hallmark of adipose tissue microenvironment proinflammatory reprogramming. Indeed, these structures can regulate immune cell infiltration by releasing free fatty acids and have been observed in many BC adipose tissues, confirming the well-acknowledged correlation between cancer and chronic inflammation [12]. Mammary adipose cells may also directly contribute to cancer progression by providing protumorigenic signals to cancer cells [13,14,15]. Furthermore, the immune cell response, as well as the inflammatory state, is directed and sustained by a hormone secreted by the adipocytes, known as leptins, whose role is hereafter further analyzed in order to define the connection between BC development and obesity-related inflammation.

## 2. Leptin Structure and Function

Leptin (LEP) is a 16 kD peptide hormone encoded by the obese (OB) gene located on chromosome 7q32.1, primarily synthesized and secreted by white adipocytes. LEP is composed of 167 amino acids and structurally presents homology sequences with the four helical cytokine subfamily that includes granulocyte colony-stimulating factor (G-CSF), interleukin-4/-5/-10 (IL-4/-5/-10), and erythropoietin (EPO) [16]. Although LEP is primarily synthesized and secreted by adipocytes, gastric, and colonic mucosa, the anterior pituitary gland, placenta, skeletal muscle, bone marrow, and lymphoid tissue play a role in its production [17,18]. LEP is known to regulate satiety by indicating the presence of sufficient energy reserves in the lateral hypothalamic area [19]. Indeed, LEP levels reflect the adipose reserve, increasing with caloric intake, and induce negative feedback on the arcuate nucleus to suppress appetite. Serum leptin levels have been correlated with adipose tissue mass, being higher in obese people than in normal-weight individuals [20].

Indeed, obese-related hyperleptinemia has been associated with leptin resistance and desensitization of leptin’s hypothalamic receptors, which leads to loss of central satiety inhibition [21].

**Figure 1:**
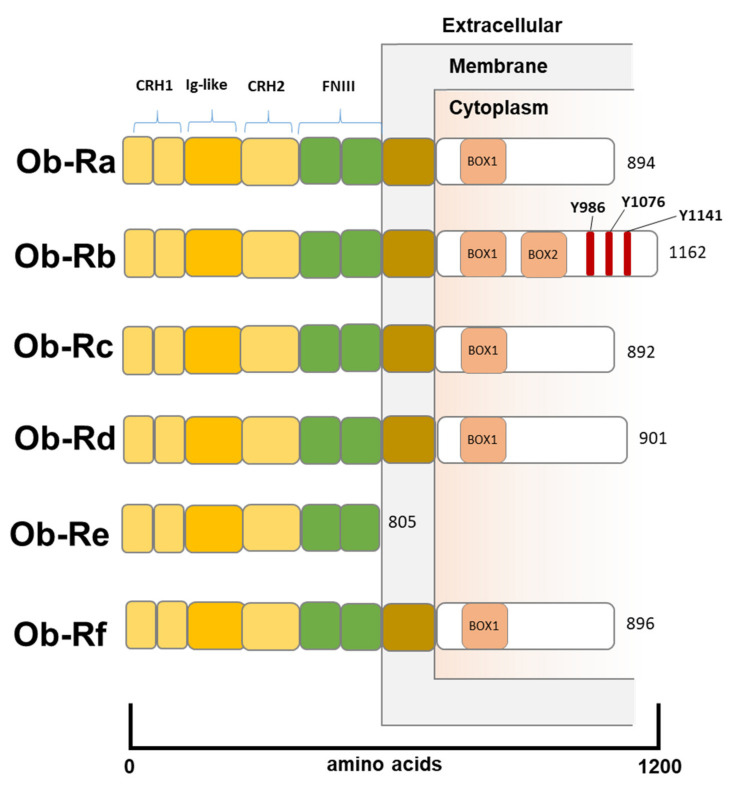
The Structure of Leptin Receptors. All leptin receptor isoforms share a binding C-terminal extracellular domain consisting of 2 CRH regions separated by an IG-like domain and followed by 2 FNIII domains. The intracellular N-terminal domain presents a BOX-1 motif, which is critical for JAK-2 binding. OB-Rb receptor is the only isoform to present a BOX-2 motif, which facilitates the activation of JAK2-STAT transduction pathway. Additionally, OB-Rb presents 3tyrosine residues (Tyr986, Tyr1076, and Tyr1141) whose phosphorylation enables STAT3-5 activation. OB-Re receptor is a soluble isoform, which binds circulating LEP and regulates LEP plasma bioavailability and functionality, not being able to transduce any downstream signal. CHR, cytokine receptor homology; IG-like, immunoglobulin-like; FNIII, fibronectin type III.

LEP functions are not limited to the hypothalamus and do not concern the maintenance of energy homeostasis [22] exclusively. Indeed, LEP is known to take part in several physiological processes such as the regulation of bone metabolism, the onset of puberty, modulation of T cells activity, stress response, maintenance of respiratory function, and blood pressure [23]. LEP interacts with a specific leptin receptor (LEPR), highly homologous to the sequences of the receptors for class I cytokines [24]. Several isoforms (OB-Ra, OB-Rb, OB-Rc, OB-Rd, OB-Re, and OB-Rf) of LEPR have been identified, resulting from alternative splicing of the *OB-R* gene. LEPR isoforms share the extracellular C-terminal leptin binding domain, while they differ in the intracellular N-terminal domain (Figure 1) [25]. The OB-Ra, OB-Rb, OB-Rc, OB-Rd, and OB-Rf isoforms present at the N-terminal domain of the BOX-1 motif, which is critical for JAK2 binding, thus leading to the activation of both the phosphatidylinositol 3-kinase/v-Akt (PI3K/AKT) pathway, via phosphorylation of the adapter protein insulin receptor substrate (IRS) and the mitogen-activated protein kinase (MAPK) pathway. OB-Rb is the main isoform responsible for the homeostatic metabolic function of LEP. OB-Rb has an N-terminal domain of 302 amino acids with an additional conservative matching motif (BOX-2), essential for the Janus kinase/signal transducer and activator of transcription (JAK/STAT) signaling pathway activation [26] (Figure 1). Ultimately, OB-Re is a soluble leptin receptor (SLE), which binds circulating LEP and regulates LEP plasma bioavailability and functionality [27] (Figure 1). 

## 3. The Role of Leptin in Breast Cancer

Accumulating evidence suggests a role for LEP in cancer development and progression by interacting with several oncogenic pathways and shaping the immune microenvironment [28].

The characterization of LEP role in BC carcinogenesis is controversial and strictly linked to the adipose endocrine function. Several studies have revealed a correlation between serum leptin levels and BC risk, suggesting that LEP may have an independent role in BC carcinogenesis [29]. A meta-analysis of 43 studies suggested a positive correlation between increased serum LEP levels and BC development. This analysis further showed that higher LEP serum levels were more commonly detected among BC patients with BMI > 25 and lymph node invasion, suggesting a potential role in promoting BC metastasis [30]. Moreover, the association of LEP levels and BC risk, beyond BMI, is strongly dependent on menopausal status. Indeed, a systematic review conducted on 35 studies identified LEP as a potential biomarker for cancer risk assessment in postmenopausal obese or overweight women, but no significant association was found in premenopausal women [31]. Interestingly, a multicenter study conducted on more than 750,000 premenopausal women reported the paradoxical effect of BMI on BC risk related to menopausal status [32]. Indeed, the analysis showed an increased risk of BC in premenopausal underweight women as compared to those who presented a BMI > 25.0. Particularly, a 4.2-fold increased risk was detected among women aged 18 to 24 years and with a BMI < 17.0 versus ≥35.0, with the association being stronger for estrogen-receptor (ER) and/or progesterone-receptor (PR) positive BC [32]. This risk imbalance, which is known to be influenced by early life events occurred before first childbirth as the peak height growth velocity, the age at menarche, adolescent physical activity, diet, and alcohol intake [33,34], has been related to estrogen serum levels, normally higher in premenopausal women. Indeed, estrogen synthesis in the premenopausal state is primarily limited to the ovaries, whereas in postmenopausal women, adipose tissue represents the main source of estrogen production through the androgens’ conversion promoted by the aromatase enzymatic complex [35]. Thus, in obese premenopausal women, the adipose tissue expansion induces negative feedback on the hypothalamic-pituitary axis, suppressing ovarian function by decreasing gonadotrophin-releasing hormone secretion (GnRH), and inhibiting ovarian estradiol production [36]. Furthermore, the hormonal deregulation eventually leads to menstrual disorders commonly associated with obesity, such as amenorrhea, impairing ovulatory cycles, and decreasing breast cell mitotic rate, which is known to be higher during the luteal phase of the menstrual cycle under estrogen and progesterone stimulation [37]. Preclinical studies have tried to shed light on the physio-pathological linkage between BC and BMI in premenopausal women. Indeed, in mouse models of 40 days of age, the administration of 17β-estradiol and progesterone has been associated with high mammary gland differentiation, providing protection against carcinogenesis subsequent to N-nitroso-N-methylurea exposure [38]. Moreover, estrogen signaling, starting early during childhood, may increase the tumor suppressor gene’s expression as BRCA1, promoting breast cell differentiation and DNA damage repair [39]. Notably, sex hormone levels may be affected by LEP activity and its binding with the long isoform receptor. In particular, LEP has been demonstrated to enhance aromatase expression in MCF-7 breast cancer cells [40], and to be strictly involved in estrogen receptor (ER) activity modulation, through the existing crosstalk between LEP and ERα [41]. In the xenografts model, indeed, it has been shown that the levels of serum estradiol double after 13 days of LEP exposure [42], whereas in MCF-7 breast cancer cell lines, estradiol administration has enhanced LEP and OB-Rb expression [43].

In addition, LEP appears to stimulate ligand-independent ER activation through the up-regulation of the mediator subunit 1 (Med1), a key coactivator transcriptional factor, via inhibition of miR-205, and increasing nuclear translocation of phosphorylated ER, thus resulting in ER transactivation and the expression of ER regulated genes. Furthermore, it has been demonstrated that Med1 may contribute to the onset of tamoxifen resistance in breast cancer cells [44] and high Med1 expression correlates with poor prognosis in tamoxifen-treated patients [45]. 

Interestingly, in addition to its involvement in ER+ BC, LEP seems to have a role in the signaling pathway transduction of HER2-positive and triple-negative BCs. Indeed, a crosstalk has been demonstrated between LEP and HER2 receptor, through HER2-OB-R physical interaction. In particular, LEP has been shown to promote HER2 transactivation and phosphorylation in either HER2 high (the expression on immunohistochemistry, IHC 3+) or low (IHC 1+/2+) expressing BC cell lines (BT-474, SK-BR-3, MCF-7, and ZR-75-1), resulting in BC cells proliferation [46]. Moreover, in triple-negative BC cell lines (MDA-MB-231, MDA-MB-468, and HCC-1806), a crosstalk between LEP and IGF-1 signaling has been demonstrated to promote the transactivation of the epidermal growth factor receptor (EGFR), which may enhance tumor cell invasion and migration [47]. Furthermore, LEP in mouse mammary cancer cells models (4T1, EMT6, and MMT) appeared to promote angiogenesis through VEGF up-regulation via canonic (JAK/STAT, MAPK, PI3K) and non-canonical (PKC, JN) signaling cascade [48]. Indeed, activated transcription factors such as HIFα and NFkβ, induced by these signaling pathways, enhance VEGF transcription, which is known to be critical for blood vessel formation from pre-existing vasculature [48]. In addition, LEP promotes VEGFR2 Tyr1175 phosphorylation resulting in endothelial cells growth and motility via COX-2 expression induced by MAPK and PI3K/AKT signaling activation [49].

**Figure 2:**
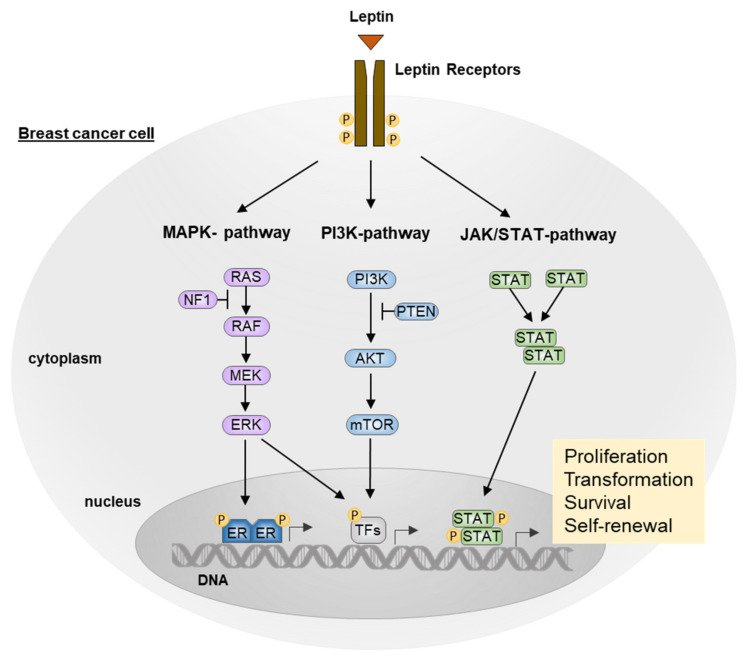
Leptin-induced oncogenic signaling pathways in breast cancer. Schematic representation of key intracellular signaling pathways associated with activation of leptin receptors upon binging with leptin in breast cancer cells. AKT, protein kinases; ER, estrogen receptor; PI3K, phosphatidylinositol 3-kinase; Ras, Rat sarcoma subfamily of GTPases; mTOR, mammalian target of rapamycin; MEK, mitogen-activated protein kinase; RAF, RAF kinase; JAK/STAT: Janus kinases/signal transducers and activators of transcription; PTEN, phosphatase and tensin homolog; TFs, transcription factors.

Hereby, we discuss in detail the oncogenic pathways that LEP, upon binding to its receptor LEPR is able to activate, thus promoting cell proliferation, migration, and angiogenesis, largely acknowledged as hallmarks of cancer [50] (Figure 2).

### 3.1. JAK/STAT Pathway

LEP binds the cytokine receptor homology site (CRH2) in the extracellular domain of LEPR. This interaction leads to receptor dimerization according to the cytokine receptor superfamily binding model [51], and the conformational change induces the cascade signaling transduction. BOX1 and BOX2 motifs in the N-terminal domain of the OB-Rb receptor lead to JAK2 activation, and the LEPR-JAK2 complex enables the phosphorylation of tyrosine residues Tyr986, Tyr1076, and Tyr1141 in the intracellular receptor domain. The phosphorylation of residues Tyr1076 and Tyr1138 enables STAT5 binding and activation, whereas phosphorylated Tyr1141 residues induce STAT3 dimerization and activation [52]. Thus, STAT3 dimers translocate in the nucleus to enhance the signaling through transcription factors such as c-jun, erg-1, and c-myc, which are involved in cancer cell proliferation [53]. Furthermore, it has been reported that LEP in breast cancer models, via JAK2/STAT3 pathway, promotes cell cycle progression by increasing the expression of cdk2 and cyclin D1 levels, thus inducing hyperphosphorylation and subsequent inactivation of the cell cycle inhibitor Rb [54]. Interestingly, in MCF-7 breast cancer cells, LEP has been demonstrated to be able to sustain, in a dose-dependent manner, the cancer cell immortalization by upregulating the expression of Human Telomerase Reverse Transcriptase (hTERT) via STAT3 dimerization [55]. Moreover, STAT3 phosphorylation promoted by LEP enhances carnitine palmitoyl transferase 1B (CPT1B) expression, which encodes the key enzyme for fatty acid β-oxidation (FAO). Thus, in BC cells derived models, FAO induced by LEP signaling seems to play a critical role in BC stem cell (BCSC) self-renewal that is associated with chemoresistance [56]. 

### 3.2. MAPK Pathway

As a result of JAK2 activation, phospho-Tyr986 residual motifs bind the Src homology 2 domain (SH2) of protein tyrosine phosphatase-2 (SHP2), which via the Growth factor receptor-bound protein 2 (GRB2) adaptor protein, leads to the ERK1/2 and JNK activation [57]. ERK nuclear translocation induces the expression of target genes, which are known to stimulate growth and cell proliferation, such as c-fos and egr-1 [57]. Furthermore, through the MAPK pathway activation, LEP promotes the growth of breast cancer in nude mice and induces the proliferation and migration of the MCF-7 BC cell line [58]. In addition, ERK1/2 activation, as well as p53 downregulation, has been demonstrated to induce aromatase expression in breast adipose stromal cells [58] and to promote functional activation of ER in the MCF-7 BC cell line [59]. 

### 3.3. PI3K/AKT Pathway

LEPR-JAK2 complex can induce IRS-1 phosphorylation, which by binding p85, the PI3K regulatory subunit, relieves p110, the catalytic subunit, thus allowing AKT activation [60]; therefore, in MCF-7 breast cancer cell lines, PI3K/AKT pathway induced by leptin has been demonstrated to promote epithelial–mesenchymal transition (EMT), which is known to facilitate cancer cells invasion, and the development of a plasticity phenotype, via IL-8 secretion [61] and pyruvate kinase M2 (PKM2) activation [62]. Furthermore, PI3K/AKT LEP-dependent signaling enables the upregulation of Acyl-CoA: cholesterol acyltransferase 2 (ACAT2) mRNA and protein expression in ER-positive BC cell lines (MCF-7, T47D, and BT474), facilitating cell migration and invasion by increasing cholesterol esterification [63].

## 4. Leptin and Immune System

**Figure 3:**
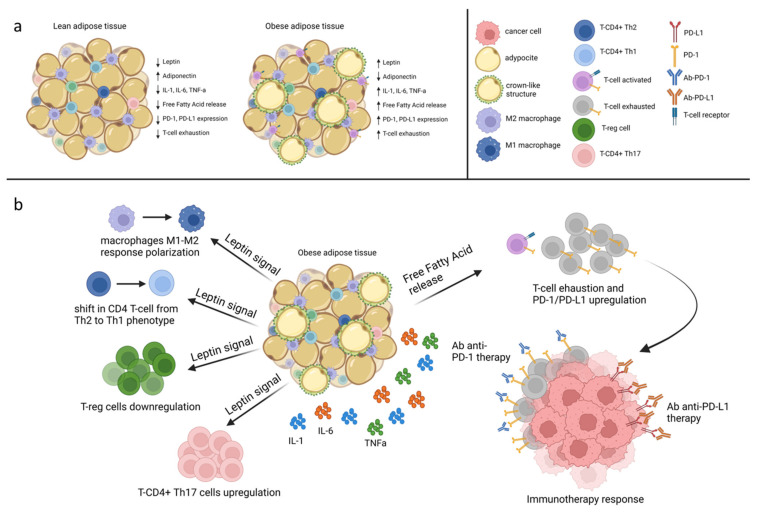
Obesity-related immune landscape. (**a**). Expansion of fat adipose tissue, compared to lean adipose tissue, is associated with a microenvironment proinflammatory state characterized by an imbalance of adipokines secretion and a systemic immune response reprogramming. Adipocytes’ hyperplasia and hypertrophy provoke adipose tissue macrophages (ATMs) activation resulting in the formation of typical crown-like structures (CLS). (**b**). Leptin increasing signal stimulates macrophages M1-M2 response polarization, induces a shift in CD4 T-cell from a Th2 towards Th1 phenotype, promotes T-reg cells down-regulation and T-CD4+ Th17 up-regulation. Free fatty acid (FFAs) release and antigenic chronic stimulation lead to T cell exhaustion and consequently PD-1 and PD-L1 upregulation, resulting in cancer cell immune escape. Eventually, PD-1/PD-L1 hyperexpression represents a paradoxical potential target for immune-checkpoint inhibitors. Figure created in BioRender (https://biorender.com/ (accessed on 15 September 2022)).

Adipose tissue expansion is known to be strongly associated with a chronic inflammatory state, resulting in a phenotypic change in adipose resident immune cells and in an imbalance of adipokines secretion (Figure 3). Indeed, as adipocyte surface area increases, a reduced thymopoiesis and T-cell progenitor pool proliferation may occur because of thymic involution, known as thymic aging, promoted by adipocyte infiltration [64]. Moreover, LEP signaling markedly increased in obese patients, resulting in several implications on T-cell function and proliferation [65]. In particular, LEP improves CD4 T-cell activation beyond the major histocompatibility complex (MHC) and induces a shift in CD4 T-cell from a Th2 towards Th1 phenotype by stimulating IL-2, IL-12, and IFN-γ expression [66,67].

Moreover, LEP has been demonstrated to influence CD4+ Th17 and CD4+ Treg differentiation. Indeed, in LEP-deficient mice (ob-ob), the exogenous hormone administration showed to enhance CD4+ Th17 upregulation, while reducing Treg proliferation [68,69]. Furthermore, in a study conducted on 30 obese and 13 lean patients, plasma LEP levels, as well as BMI, resulted in being inversely correlated with Treg cells circulating numbers in the obese group, whereas no correlation was observed in the control group [70]. In addition, LEP levels were observed to be positively associated with CD4+ Th17 circulating percentage in patients diagnosed with autoimmune disease as Hashimoto’s thyroiditis [71] and negatively with CD4+ CD25+ regulatory cells observed in relapsing-remitting multiple sclerosis (RRMS) patients [72]. 

LEP activity also exerts an impact on the innate immune response. Indeed, LEP promotes IL-1, IL-6, and TNFα secretion by monocytes and induces an M1 pro-inflammatory macrophage response polarization through mast cells (MCs) signaling [73]. Indeed, in ob-ob mice models, MCs resident in white adipose tissue showed to drive an anti-inflammatory macrophage phenotype (M2), whereas MCs depletion has been proven to facilitate weight gain and obesity [73].

Additionally, the knockdown of LEPR in ER-positive MCF-7 and in triple-negative MDA-MB-231 BC cells (ObR sh) was demonstrated to hamper macrophage recruitment in the tumor microenvironment (TME) and influence immune response. In particular, in mice models injected with MCF-7 and MDA-MB-231 ObR sh clones, a reduced tumor-associated macrophage (TAMs) infiltration within xenograft tumors was observed. Moreover, in line with the decreased macrophage recruitment, a lower expression on TAMs surface of programmed cell death protein 1 (PD-1), commonly associated with cancer immune escape, was detected [74].

This obesity-related “meta-inflammatory” state [75,76] can eventually impair antitumor immunity: in addition to increasing Treg differentiation and promoting M1 proinflammatory macrophages polarization, it can also act by exhausting immune response through low inflammatory chronic stimulation of Toll-like receptors (TLRs) promoted by lipolysis and secretion of FFAs [77].

The immune regulation promoted by LEP in the inflamed adipose tissue may influence BC carcinogenesis and treatment response. Indeed, in a study conducted on 87 breast cancer patients treated with neoadjuvant chemotherapy, LEP and LEPR overexpression, were found in patients with higher levels of tumor-infiltrating lymphocytes (TILs). This association was observed mainly in patients with HER2+ BC (21.5% vs. 9%; *p* = 0.015) and, among those with high levels of LEPR, in patients who achieved a complete pathological response compared to those with residual disease (26.6% vs. 12.5%; *p* = 0.005) whereas this imbalance was not observed in patients with the LEPR not overexpressed [78].

Furthermore, a retrospective analysis conducted on 445 TNBC patients treated with neoadjuvant chemotherapy evaluated the response rate and TILs percentage according to BMI. High TILs were significantly associated with pathological complete response (pCR) in lean patients (OR = 4.24, 95% CI = 2.10 to 8.56; *p* < 0.001) but not in overweight and obese patients (OR = 1.48, 95% CI = 0.75 to 2.91; *p* = 0.26) [79].

Interestingly, despite facilitating cancer immune evasion, obesity-related T cell exhaustion, occurring as a result of thymic aging and chronic antigenic stimulation [65,76], leads to the compensatory increase in PD-1/PD-L1 expression on immune cells, providing a potential target for immune-checkpoint inhibitors [80].

Indeed, the tumorigenic immune dysfunction appeared to be reversed by anti-PD-1/PD-L1 treatment in a retrospective analysis conducted on 976 advanced cancer patients stratified according to BMI. The analysis included 635 patients diagnosed with NSCLC, 183 patients with melanoma, 135 patients with renal cell carcinoma, and 23 with other tumors [81]. In particular, overall response rate (ORR) and time to treatment failure (TTF) were found to be significantly higher in overweight/obese patients (BMI ≥ 25.0), compared to non-overweight (*p* < 0.0001), as well as median progression-free survival (PFS) being longer in the first group (11.7 months vs. 3.7 months; HR= 0.46 *p* < 0.0001). Furthermore, these results are consistent with those observed in a retrospective analysis which included patients (*n* = 2046) diagnosed with metastatic melanoma stratified according to BMI and treated with targeted therapy, immunotherapy, or chemotherapy. Indeed, obesity compared with normal BMI was associated with improved progression-free survival (PFS) and overall survival (OS) in the cohort of patients treated with immunotherapy and target therapy [82].

Despite the absence of available data on obese BC patients, in obese female mice fed with an obesogenic high-fat diet, injected with BC E0771 cells and treated with anti-PD-1 therapy, it has been demonstrated a significant decrease in tumor volume, a reduced cell proliferation and a concomitant elevation of CD8+ T cells, CD4+ Th1 cells and dendritic cells [83].

## 5. Leptin Signaling as a Potential Target for Therapeutic Intervention

As LEP signaling transduction is a well-known carcinogenesis promoting factor, diverse therapeutic agents have been designed to modulate the LEP cascade. Great research efforts focused on the development of LEPR antagonists able to interfere with LEP binding activity. The OB-R antagonist Allo-aca peptide, an analog of OB-R leptin binding site III, has shown promising activity in delaying BC progression and extending survival in TNBC mice models; however, its use was associated with an accelerated weight gain [84]. The peptide LDFI (Leu-Asp-Phe-Ile), a selective LEPR antagonist, was developed based on leptin binding site I and demonstrated to inhibit the growth and motility of both ER-positive MCF-7 and ER-negative SKBR3 breast cancer cell lines. Notably, the PEGylated isoform of LDFI (PEG-LDFI) showed comparable antitumor efficacy in xenograft models without affecting energy balance, since no changes in body weight were observed [85]. In addition, the peptide LDFI recently showed to reduce cell-to-cell communication by decreasing LEP-dependent exosome biosynthesis in MCF-7 and triple-negative MDA-MB-23 breast cancer models [86]. Honokiol (HNK) is a bioactive polyphenol from magnolia grandiflora, acting as a leptin antagonist. HNK is demonstrated to reduce LEP-induced EMT by targeting the STAT3/Zeb1/E-cadherin axis [87]. However, despite the attractive results from several preclinical studies, no clinical trials evaluating LEP antagonists in patients with BC have been conducted to date.

Alternative strategies to target LEP-associated signaling have been explored. The 1,25(OH)2D3 showed to downregulate the human telomerase reverse transcriptase (hTERT) activation promoted by LEP in both breast and ovarian cancer models [88]. Since LEP induces CPT1B expression and FAO activity in BC stem cells to ensure self-renewal and chemoresistance, FAO inhibitors such as perhexiline were able to restore chemosensitivity in BC cells [56]. LEP signaling also enhances aromatase expression [43], impairing tamoxifen treatment efficacy [47,48]. Therefore, aromatase inhibitors (AIs) based regimen exhibits the major beneficial effects on the LEP target cascade. Finally, PD-L1/PD1 upregulation enhanced by the obese-related low chronic inflammatory state could be exploited to improve the immune checkpoint inhibitors treatment activity, especially in patients diagnosed with TNBC, which is known to be the most immunogenic breast cancer subtype.

## 6. Conclusions

Leptin is a peptide hormone known to play a critical role in the meta-inflammatory state observed in obesity. Many mechanisms appear to be involved in the correlation between obese-related LEP activity and breast cancer carcinogenesis. LEP signaling cascade promotes breast cancer cell proliferation, migration, and invasion through the activation of MAPK, PI3K/AKT, JAK2/STAT pathways, and EMT, irrespective of BC subtypes, primarily in post-menopausal women. Moreover, LEP is involved in immune activity exhaustion associated with low chronic inflammation in obese cancer patients, which could be employed to improve the response to immune checkpoint inhibitors. Novel therapeutic strategies, such as LEP or LEPR antagonist, are not currently under development, although they would represent a promising step toward a more personalized treatment approach for breast cancer patients.
